# Targeting Tumour-Initiating Cells with TRAIL Based Combination Therapy Ensures Complete and Lasting Eradication of Multiple Myeloma Tumours *In Vivo*

**DOI:** 10.1371/journal.pone.0035830

**Published:** 2012-05-16

**Authors:** Srdjan Vitovski, Andrew D. Chantry, Michelle A. Lawson, Peter I. Croucher

**Affiliations:** 1 The Mellanby Centre for Bone Research, Department of Human Metabolism, University of Sheffield Medical School, Sheffield, United Kingdom; 2 Department of Infection and Immunity, The Medical School, Sheffield, United Kingdom; 3 Garvan Institute for Medical Research, Sydney, Australia; The University of Birmingham, United Kingdom

## Abstract

Multiple myeloma (MM) remains an incurable disease despite improvements to available treatments and efforts to identify new drug targets. Consequently new approaches are urgently required. We have investigated the potential of native tumour necrosis factor-related apoptosis-inducing ligand (TRAIL), in combination with doxorubicin, to induce apoptotic cell death in phenotypically distinct populations of myeloma cells *in vitro* and *in vivo*. The cytotoxic potential of TRAIL alone, and in combination with DOX, was assessed *in vitro* in purified CD138^+^ and CD138^−^ cells from the MM cell lines and samples from patients with MM. Mouse xenografts obtained by implanting CD138^−^ MM cells were used to assess the efficacy of TRAIL, alone and in combination with DOX, *in vivo*. CD138^−^ cells were shown to be more resistant to the cytotoxic activity of TRAIL than CD138^+^ cells and have reduced expression of TRAIL death receptors. This resistance results in preferential killing of CD 138^+^ cells during exposure of MM culture to TRAIL. Furthermore, prolonged exposure results in the appearance of TRAIL-resistant CD138^−^ cells. However, when TRAIL is combined with doxorubicin, this results in complete eradication of MM cells *in vivo*. Most importantly, this treatment successfully eliminates CD138^−^ cells implicated in tumour initiation and growth maintenance. These findings may explain the failure of current therapies and offer a promising new approach in the quest to cure MM and disseminated cancers.

## Introduction

Multiple myeloma (MM) is a clonal B cell malignancy characterised by the accumulation of plasma cells in the bone. It is the second most prevalent blood cancer and accounts for approximately 10% of all haematological cancers [1]. MM is characterised by the development of osteolytic lesions, anaemia and immunosuppression.

MM cells are typically found in multiple bone marrow sites. Thus, only systemic treatments, such as chemotherapy or irradiation are likely to result in substantial tumour reduction and remission. However, despite developments in treatment, current strategies have not been successful in the complete eradication of malignant plasma cells, and MM remains an incurable disease. Median survival with conventional chemotherapy (malphalan+prednisone) regimens is approximately 3 years [2] and more aggressive chemotherapy is associated with only a modest improvement [3], [4]. High-dose therapy with stem cell transplantation improves response and long-term remission in younger patients, but results in only a modest survival advantage compared to conventional therapy [5]. More recently, the introduction of new, targeted, therapies such as lenalidomide and bortezomib, has resulted in increased survival, however, even with these new agents patients ultimately relapse [6], [7]. In addition, treatment with some chemotherapeutic drugs is associated with the appearance of secondary malignancies [8]–[10] suggesting that new approaches are urgently required.

Recent advances in our understanding of the origin and development of malignant transformation has highlighted the importance of tumour-initiating cancer “stem” cells. These cells may explain the failure of current approaches to cancer treatment [11], [12]. An important feature of these cells is their relative quiescence. Conventional chemotherapeutic agents often target rapidly dividing cancer cells and leave normal cells unharmed. It follows that the differentiated, rapidly dividing cancer cells, which represent the majority of cells within the tumour, are comparatively easy to treat when compared to the slow dividing tumour-initiating cells. Furthermore, survival and development of drug resistance in this population could further limit future therapeutic options. The existence of these cells has been reported in a variety of cancers, including acute myeloid leukaemia, breast, prostate and myeloma [13]–[16].

Early work in MM has demonstrated that only a small proportion of cells are capable of clonogenic colony-forming growth [17]. The hypo-proliferative nature of myeloma has led to the hypothesis that the bulk of the tumour results from a transformed B cell with the capacity for both self-renewal and production of terminally differentiated progeny [18]. Recently, it has been shown that clonogenic progenitor cells can be successfully isolated from established MM cell lines as well as from patient bone marrow samples [16]. Furthermore, it has been reported that syndecan 1 negative cells (CD138^−^), which represent only a minor fraction of MM cells, possess the capacity for unlimited self-renewal *in vitro* and the capacity to induce tumour growth *in vivo* [16].

There is increasing evidence to indicate that the process of apoptosis plays a central role in tumour establishment and resistance to chemotherapy [19]–[23]. It has been suggested that anticancer drugs that induce apoptosis in tumour cells by activation of the death receptor pathway may offer an improved treatment for patients with MM [24]–[26]. Tumour necrosis factor-related apoptosis-inducing ligand (TRAIL/Apo2L) belongs to the tumour-necrosis factor (TNF) super-family of related proteins [27]. TRAIL functions as a homotrimer and mediates its biological activity through four TRAIL receptors [28]. Two of these receptors, TRAIL R1 and TRAIL R2, contain death domain motifs and promote apoptosis. TRAIL binding to R1 or R2 results in signalling through the death-inducing signalling complex and induces apoptosis in tumour cells but not normal cells [29]–[30], suggesting that one of the normal physiological roles of TRAIL is in immune surveillance [31]–[33]. Importantly, it has been shown that myeloma cells can develop resistance to TRAIL [34]–[35]; however, conventional chemotherapeutic drugs can sensitise these resistant cells to TRAIL through up-regulation and activation of death receptors R1 and R2 [35]–[36]. Indeed, the anthracycline antibiotic, doxorubicin (DOX) has been shown to have the greatest potential for sensitisation of tumour cells to TRAIL [37]–[38]. Whether all cells develop resistance to TRAIL or whether this is limited to those with tumour-initiating potential is unclear.

We have previously reported the expression and purification of native human TRAIL in *E. coli* [39]. Furthermore, we, and others, have shown that TRAIL induces apoptosis in human multiple myeloma cells [34]–[36], [40], [41]. However, it is unclear whether TRAIL has similar effects in both CD138^+^ and CD138^−^ populations and whether combination with chemotherapeutic agents increases the sensitivity of myeloma cells to TRAIL. Therefore, the aim of the present study was to determine whether recombinant human TRAIL could induce apoptosis in CD138^+^ and CD138^−^ cells and whether TRAIL in combination with DOX could cause sustained eradication of human MM cells *in vitro* and *in vivo*.

## Materials and Methods

### Ethics statement

Bone marrow samples were obtained from patients with multiple myeloma after obtaining informed written consent (South Sheffield Research Ethics Committee (SSREC) reference 05/Q2305/96). All treatment protocols involving animals were approved by the UK Home Office (project licence PPL 40/2901).

### Reagents

TRAIL was expressed and purified as we previously described [39]. Doxorubicin and dexamethasone were purchased from Sigma (Poole, UK). Reagents were dissolved in culture media such that identical volumes were used within experiments.

### Cell culture

The human MM cell lines, RPMI8226 and NCI H929 were obtained from the American Type Culture Collection (Rockville MD, USA) and the OPM-2 line from the German Collection of Microorganisms and Cell Cultures (Braunschweig, Germany). Cells were maintained in RPMI 1640 medium (Life Technologies) supplemented with 10% foetal bovine serum, 1% penicillin/streptomycin, 1% sodium pyruvate, and 1% nonessential amino acids. Cells were incubated at 37°C, with saturated humidity and an atmosphere of 5% CO_2_.

### Patient samples

Bone marrow samples were obtained from patients (3 male, 2 female, mean age 70.2 years), with newly diagnosed multiple myeloma, after obtaining informed written consent (SSREC reference: 05/Q2305/96). These patients had not previously undergone treatment. The mononuclear cell fraction was isolated by density gradient centrifugation over Lymphoprep according to manufacturer instructions (Nycomed Pharma, Oslo, Norway).

### Separation of CD138^+^ and CD138^−^ cells

Purification was performed as described previously [16]. Briefly, CD138^+^ cells were obtained from cultures of RPMI8226, NCI H929 or OPM2 cells, or patient mononuclear cell samples, by immunomagnetic separation. Cells were incubated with microbeads conjugated with anti-human CD138 antibodies and separated using a magnetic column (Miltenyi Biotec, Bisley, UK). CD138^−^ cells were obtained after depletion of CD138^+^ cells from the culture. CD138^−^ cells isolated from patient bone marrow samples were defined as cells within the fraction that were CD138^−^ and CD34^−^. CD138^−^CD34^−^ cells were obtained after immunomagnetic depletion of the CD138^−^ mononuclear fraction of normal haematopoietic cells using mouse anti-human CD34 antibodies coupled to microbeads (Miltenyi Biotec).

### Determining the cytotoxic effects of TRAIL, doxorubicin (DOX) and dexamethasone (DEX) on CD138^+^ and CD138^−^ myeloma cells

The ability of TRAIL, DOX, DEX or TRAIL in combination with DOX to reduce cell viability was determined using a colorimetric assay as reported previously [39]. Briefly, each compound was diluted in 50 µl of RPMI1640 culture media and added to the wells of a 96-well tissue culture plate containing CD138^+^ or CD138^−^ cells (2×10^5^ per 50 µl) and incubated for 48 h at 37°C. Twenty microlitres of CellTiter96^R^ Aqueous One Solution Reagent containing 3-(4,5-dimethylthiazol-2-yl)-5-(3-carboxymethoxyphenyl)-2-(4-sulfophenyl)-2H-tetrazolium (MTS) and phenazine ethosulfate (PES) (Promega, Madison, WI) were added to each well. Absorbance at 490 nm was measured after 2 or 4 h. Wells containing cells only and wells containing medium only were used as controls.

### Cell preparation and staining for microscopic examination

RPMI 8226 cells were washed in PBS and 0.1×10^5^ cells re-suspended in 0.2 ml of PBS and cytospun onto glass slides. Cells were fixed in 0.2 ml of 4% paraformaldehyde for 10 minutes at room temperature and washed again in PBS (×2). Cells were stained with haematoxylin and eosin, dehydrated through ethanol and xylene, mounted with DPX (Fisher Scientific, UK) and examined under light microscopy using ×40 objective.

### Assessment of apoptosis by annexin V labelling and flow cytometric analysis

The ability of TRAIL to induce apoptosis of myeloma cells was assessed by flow cytometric analysis of APC-conjugated annexin V and 7-aminoactinomycin D (annexin V-APC/7 AAD) stained cells. Cells (1×10^5^/ml) were grown for 18 hours and then left untreated or treated with TRAIL (100 ng/ml) for 6 hours. Cells were washed twice in PBS and labelled with annexin V-APC/7 AAD for 15 minutes and immediately analysed by flow cytometry. Cells negative for both 7AAD and annexin V-APC staining are live cells, 7AAD negative, annexin V-APC positive cells are early apoptotic cells, whereas, 7AAD-positive, annexin V-APC-positive stained cells are cells in the late stages of apoptosis or dead.

The effect of DOX (500 ng/ml) on apoptosis of myeloma cells treated cells was assessed by fluorescein isothiocyanate-conjugated annexin V and TO-PRO-3 iodide (annexin V-FITC/TOPRO 3) labelling to avoid a DOX interference with the detection of annexin V-APC/7 AAD labelling.

### Measurement of caspase activity

Caspase 3+Caspase 7 activity was determined using the Caspase-Glo® 3/7 Assay (Promega, Madison, WI) according to manufacturer's instructions. The assay provides a luminogenic caspase 3/7 substrate, which contains the caspase recognisable tetrapeptide sequence DEVD. Addition of Caspase-Glo® 3/7 reagent results in cell lysis, followed by caspase cleavage of the substrate and release of aminoluciferin, a substrate for luciferase. The luminescence produced is proportional to the amount of caspase activity present. For each assay sample 1×10^5^ cells per millilitres were aliquoted into a single well of a 24-well plate and maintained for 18 hours. RPMI8226, NCI H929 and OPM2 cells were treated with DOX (50 and 500 ng/ml for 6 hours), TRAIL (100 ng/ml for six hours) or with DOX (50 ng/ml) for 12 hours followed by TRAIL treatment (10 ng/ml for 6 hours). Twenty-five microlitres of each samples was transferred into a single well of a 96-well plate and 25 microlitres of Caspase-Glo® 3/7 Assay mixture added. After incubation for 30 minutes at 22°C luminescence was measured using a SpectraMax 5 luminometer/plate reader. All treatments were performed in triplicate and the measured luminescence signal (in luminescence relative units or LRU) was used as a direct measurement of caspase activity. Background readings were determined from wells containing culture medium alone and subtracted from each sample reading.

Caspase 8 and caspase 9 activity was determined using the Caspase-Glo® 8 and the Caspase-Glo® 9 Assays, respectively, (Promega, Madison, WI) according to manufacturer's instructions and the above protocol.

### Measurement of the effect of TRAIL on the relative ratio of CD138^+^ and CD138^−^ cell populations in culture

Exponential cultures of RPMI8226 cells were divided into two aliquots. In one aliquot CD138^+^ and CD138^−^ cells populations were separated as described above and cell number determined. The second aliquot was treated with TRAIL (25 ng/ml) and incubated for a further 48 h. CD138^+^ and CD138^−^ cells were then isolated using immunomagnetic separation and cell numbers determined. Data are expressed relative to the untreated samples.

### Generation of TRAIL-resistant CD138^+^ and CD138^−^ cells *in vitro*

RPMI8226 cells were grown in the presence of TRAIL (25 ng/ml). Surviving cells were transferred to a fresh medium containing TRAIL every 5 days. After 10 cycles of treatment the CD138^+^ and CD138^−^ cells were isolated by immunomagnetic separation. The cytotoxic activity of TRAIL was determined on CD138^+^ and CD138^−^ cells isolated from TRAIL-resistant culture and compared to CD138^+^ and CD138^−^ cells isolated from parental TRAIL-sensitive RPMI8226 culture.

### Measurement of TRAIL receptor expression

Separated CD138^+^ and CD138^−^ RPMI8226, NCI H929 and OPM2 cells were incubated with mouse anti-human TRAIL receptor R1 and R2 phycoerythrin (PE)-conjugated monoclonal antibody. Flow cytometric analysis was performed on a FACSCalibur (BD Biosciences, Oxford, UK) using the Cellquest (acquisition) and the Flowjo (data analysis) software packages.

### Determining the effect of TRAIL, DOX and TRAIL in combination with DOX on myeloma cell growth *in vivo*

All studies were performed using a non-obese diabetic/severe combined immunodeficiency (NOD/SCID) mouse xenograft model. Female mice (8–10 weeks of age) were first irradiated with 300 Rad using a ^137^Cs irradiator. Separated CD138^−^ RPMI8226-GFP cells (1×10^6^ in 0.1 ml PBS) were prepared as described above and were injected subcutaneously into the right flank of NOD/SCID mice 24 h post irradiation. After formation of palpable tumours mice were randomised into four groups and treated each day for five consecutive days by subcutaneous injection with one of four treatments. The first group (control, n = 5) received 100 µ PBS only, the second (DOX, n = 5) received DOX (1 mg/kg) and the third (TRAIL, n = 5) TRAIL (10 mg/kg). The fourth group (DOX+TRAIL, n = 10) received DOX on the first and third day only (1 mg/ml) and TRAIL (10 mg/kg) on each day.

Body weight was monitored continuously and used as an indicator of systemic toxicity and general health status. Tumour growth was assessed by measuring tumour size on digitalised images of tumours taken by LIGHTool imaging system (Lightools Research, CA, USA) at regular intervals. Each picture of tumour was first magnified to facilitate subsequent manipulations. The IMAGEJ software (http://rsb.info.nih.gov/ij/) was used to calculate size and average brightness of the tumour area. The product (area size multiplied by average area brightness) was used as a measure of the tumour size of the particular tumour at the specific time point. The mean value for tumour size for each experimental group at a given time point was calculated. Tumour growth was monitored on each day during the treatment week and then every second day for the following three weeks. Mice in the PBS, DOX and TRAIL group were sacrificed at day 55 after tumour cell inoculation. At sacrifice, tumour burden was measured by the LIGHTools imaging system and individual organs examined for evidence of tumour growth. Mice in group four (TRAIL plus DOX combination) were monitored for at least 90 days from the start of the treatment.

### Statistical analysis

Comparisons between groups were determined by Student's *t*-test or analysis of variance as appropriate (GraphPad Prism software). All data represent the mean ± standard deviation.

## Results

### CD138^+^ RPMI8226 cells are more sensitive to TRAIL than to DOX and DEX

**Figure 1:**
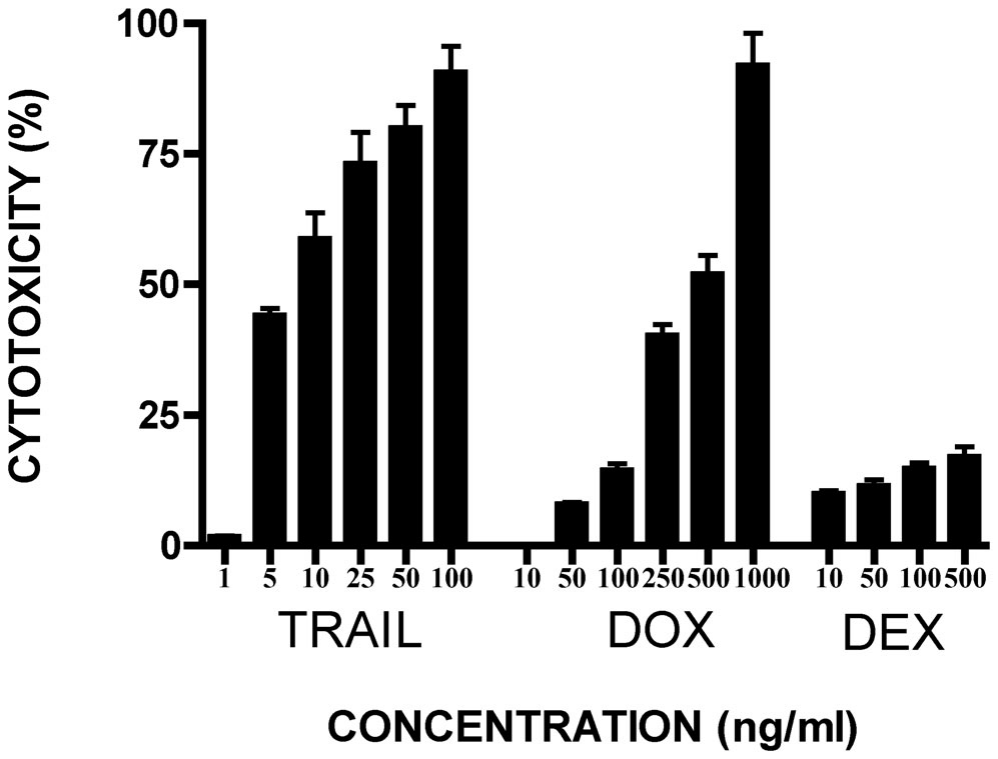
*In vitro* sensitivity of the purified CD138^+^ RPMI8226 cells to DOX, TRAIL and DEX. After immunomagnetic separation of CD138^+^ RPMI8226 cells from the parent population, cells (1×10^5^ /ml) were incubated with increasing concentrations of TRAIL (1–100 ng/ml), Doxorubicin (DOX, 10–1000 ng/ml), or Dexamethasone (DEX, 10–500 ng/ml) for 48 h. DEX was included as a positive control. Cytotoxicity was measured by MTS assay and expressed as a percentage of the untreated control sample. For details see Materials and Methods. Data represent the mean ±1SD of the three independent experiments.

To determine the activity of our native TRAIL preparation we compared the sensitivity of CD138^+^ RPMI8226 cells, isolated from the parent population, to increasing concentrations of TRAIL and compared it to that of DOX or DEX using an MTS cytotoxicity assay (Fig. 1). CD138^+^ RPMI8226 cells represent 97–99% of the unselected population and have similar characteristics to the unselected population, i.e. treatment with TRAIL or DOX results in similar levels of cytotoxicity. DEX was included as a positive control as this compound is used in clinical practice to treat MM. Treatment of CD138^+^ cells with TRAIL, DOX or DEX was associated with a dose-dependent increase in cytotoxicity (p<0.0001 for TRAIL and DOX and p<0.0129 for DEX) (Fig. 1). The cytotoxic effect of TRAIL was more pronounced, causing a 50% reduction in cell viability at a concentration of 5 ng/ml. In contrast, doses of 500 ng/ml of DOX were required to induce 50% cytotoxicity of CD138^+^ cells. DEX had only a modest ability to reduce viability of CD138^+^ cells as concentrations as high as 500 ng/ml were only able to induce 20% cytotoxicity.

### Sensitivity of CD138^+^ RPMI8226 cells to TRAIL is increased by pre-incubation with doxorubicin

**Figure 2:**
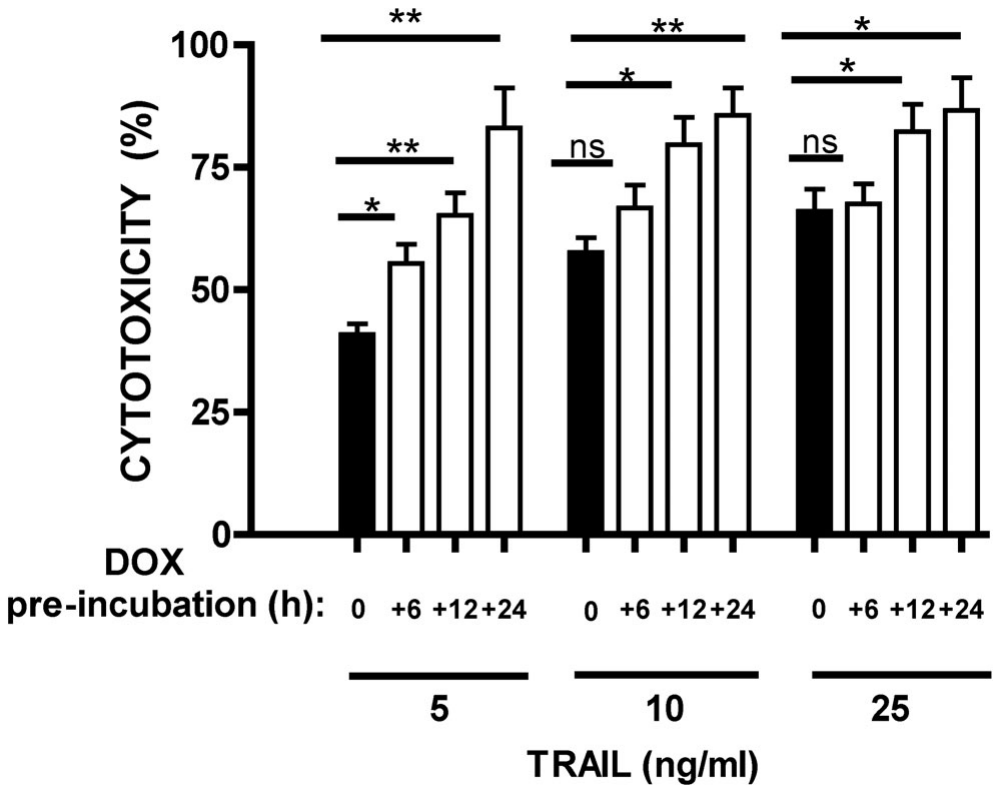
*In vitro* sensitivity of CD138^+^ RPMI8226 cells to TRAIL alone, or TRAIL after pre-incubation with DOX. Following immunomagnetic separation, CD138^+^ cells (1×10^5^ /ml) were incubated with increasing concentrations of TRAIL alone (5, 10 and 25 ng/ml) for 24 h (black bars), or TRAIL for 24 h after initial pre-incubation with DOX (500 ng/ml) for 6, 12 or 24 h (white bars). Cytotoxicity was measured by MTS assay and expressed as a percentage of the untreated control sample. For details see Materials and Methods. Data represent the mean ±1SD of the three independent experiments.

Since both TRAIL and DOX were shown to dose-dependently increase cytotoxicity of CD138^+^ cells we chose to establish whether DOX could increase their sensitivity to TRAIL. Purified CD138^+^ RPMI8226 cells were incubated with increasing concentrations of TRAIL alone (5, 10 and 25 ng/ml) after pre-incubation with DOX (500 ng/ml) for varying times (Fig. 2). This pre-incubation regime was chosen as this approach has been shown to have a higher potential to sensitise cells to TRAIL than when both agents are being given simultaneously [38]. Pre-incubation was performed for 6, 12, or 24 h and incubation with TRAIL continued for an additional 24 h. (Fig. 2). DOX significantly augmented TRAIL cytotoxicity at all TRAIL concentrations used when compared to TRAIL alone. The magnitude of augmentation increased with duration of the pre-incubation period. The highest relative increase in cytotoxicity was detected at the lowest concentration of TRAIL (5 ng/ml).

### Apoptotic cell death is the mechanism of TRAIL-induced cytotoxicity

**Figure 3:**
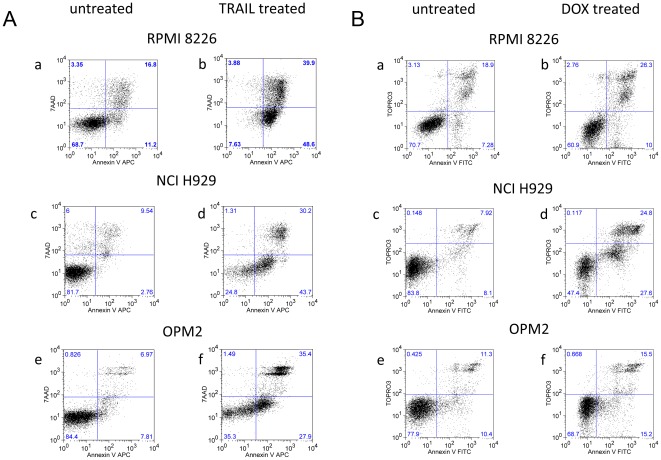
Flow cytometric analysis of Annexin V labelled myeloma cells after treatment with TRAIL or DOX. The RPMI8226, NCI H929 and OPM2 cells (1×10^5^/ml) were grown for 18 hours and then left untreated or treated with TRAIL (100 ng/ml) (3A) or DOX (500 ng/ml) (3B) for 6 hours. Cell death was assessed by annexin V staining and cellular uptake of either 7AAD for TRAIL treatment (Fig. 3A) or TOPRO3 for DOX treatment (Fig. 3B). *In vitro* apoptosis of myeloma cells was assessed by flow cytometric analysis of annexin V-APC/7 AAD stained cells (TRAIL treated) or annexin V-FITC/TOPRO3 stained cells (DOX treated).

Assessment of apoptosis in RPMI8226, NCI H929 and OPM2 cells was determined by annexin V labelling and flow cytometric analysis. Apoptosis of TRAIL treated myeloma cells was assessed by flow cytometric analysis of APC-conjugated annexin V and 7-aminoactinomycin D (annexin V-APC/7 AAD) staining (Fig. 3A). The cells (1×10^5^/ml) were grown for 18 hours and then left untreated or treated with TRAIL (100 ng/ml) for 6 hours. Cells negative for both 7AAD and annexin V-APC staining are live cells (the number in the bottom left quadrant indicate the percentage), 7AAD negative, annexin V-APC positive staining cells are early apoptotic cells (bottom right quadrant), 7AAD-positive, annexin V-APC-positive staining cells are cells in the late stages of apoptosis or dead (top right quadrant). TRAIL-induced apoptosis in all three cell lines (Fig. 3).

DOX treated (500 ng/ml) cells were assessed by fluorescein isothiocyanate-conjugated annexin V and TO-PRO-3 iodide (annexin V-FITC/TOPRO 3) labelling to avoid a strong DOX interference detected with the annexin V-APC/7 AAD labelling (Fig. 3B). DOX induced apoptosis in all three cell lines, however the level of induction was lower compared to TRAIL-induced apoptosis.

**Figure 4:**
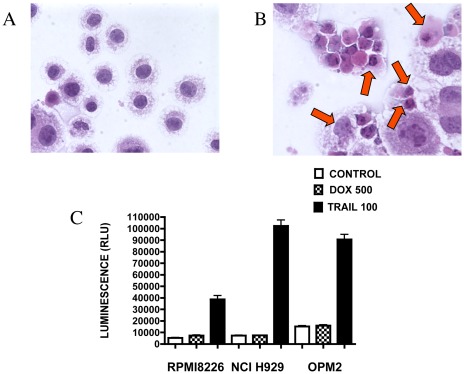
Morphological changes and caspase activity in RPMI8226 cells undergoing apoptotic cell death after exposure to TRAIL. Cells (1×10^5^/ml) were grown for 18 hours and then left untreated or treated with TRAIL (100 ng/ml) for 6 hours. Untreated (A) and TRAIL-treated cells (B) were cytospun onto microscopic slides, stained with haematoxylin (nuclei) and eosin (cytoplasm) and examined under light microscopy using ×40 objective. Apoptotic cells are indicated by an arrow. (C) Induction of effector caspase3+effector caspase 7 activities in RPMI8226 myeloma cells after treatment with TRAIL or DOX. The cells (1×10^5^/ml) were grown for 18 hours and then left untreated or treated with TRAIL (100 ng/ml) or DOX (500 ng/ml) for 6 hours. Data represent the mean ±1SD of the three independent experiments.

Microscopic examination of haematoxylin and eosin stained cells revealed the morphological differences between untreated and TRAIL-treated RPMI8226 cells. Untreated cells had defined rounded nuclei and uniformly stained cytoplasm (Fig. 4A). In contrast, TRAIL-treated cells showed typical signs of apoptosis with condensed and fragmented nuclei and blebbing or detaching of cytoplasm (Fig. 4B).

To confirm exposure to TRAIL results in apoptosis we measured the activity of caspase 3 and caspase 7, enzymes involved in apoptotic signal transduction. TRAIL treatment resulted in an induction of caspase activity when compared to untreated control cells in each line (p<0.001) (Fig. 4C). In contrast, DOX treatment had no effect on caspase activity in NCI-H929 and OPM2 cells and only a modest effect in RPMI8226 (p<0.05).

### TRAIL induces myeloma cell death by activating both extrinsic and intrinsic pathways of apoptosis

**Figure 5:**
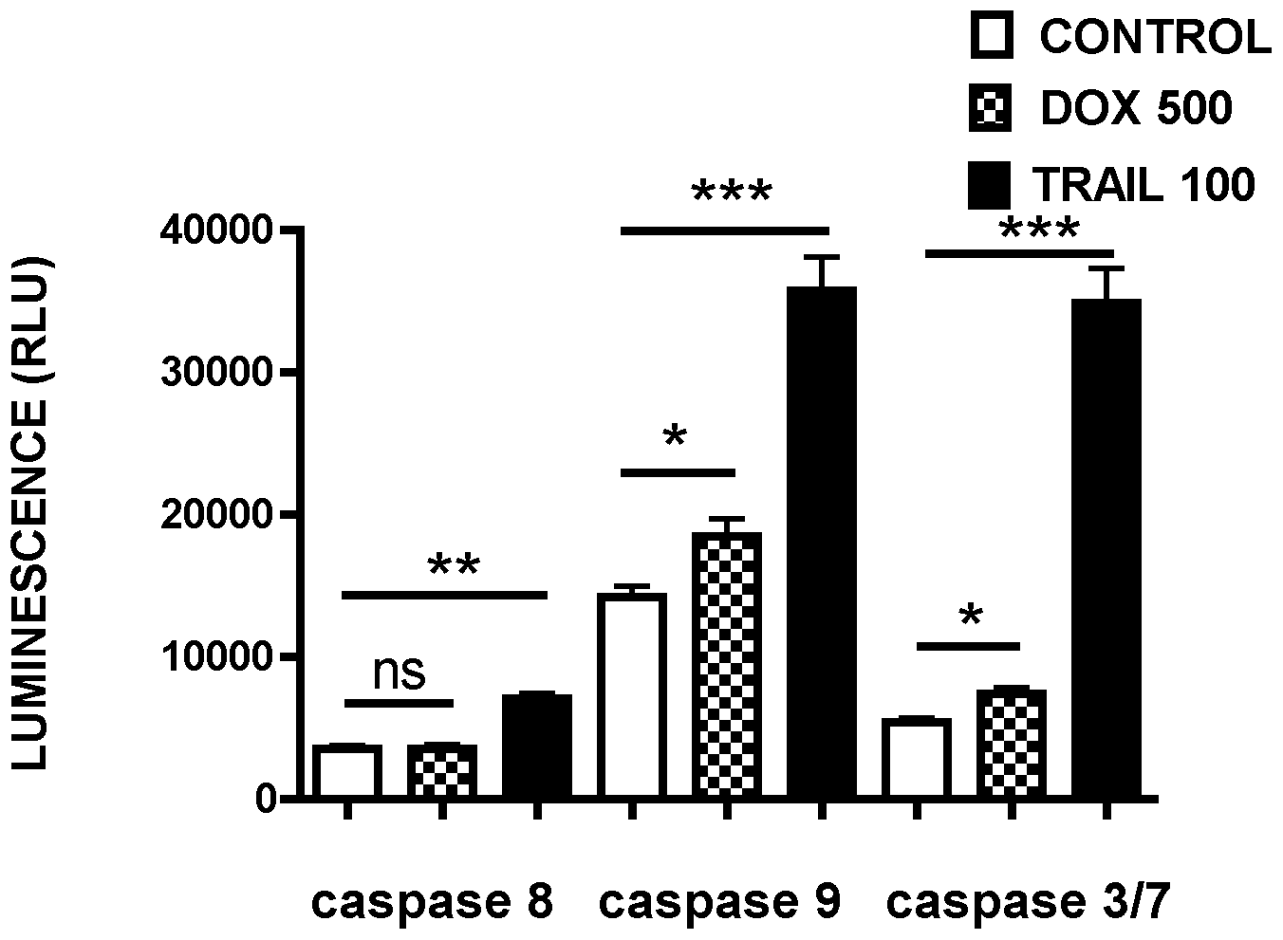
Initiator Caspase 8 and initiator caspase 9 activity in RPMI 8226, NCI H929 and OPM2 cells after TRAIL or DOX treatment. Cells were grown for 18 hours and treated with DOX (500 ng/ml) or TRAIL (100 ng/ml) for 6 hours or left untreated as control samples. The caspase-Glo® 3/7, caspase-Glo® 8 or caspase-Glo® 9 Reagent (50 µl) was added directly to each well of 96-well plate containing 50 µl of cells. The plate was incubated at 22°C and luminescence was recorded after 30 minutes. Caspase activities were expressed in relative luminescence units (RLU) as direct readings from the luminometer. Data represent the mean ±1SD of the three independent experiments. * = p<0.05, **p<0.01, ***p<0.001.

To determine the molecular mechanism of TRAIL and DOX induced apoptosis the activity of caspase 8 (early initiator caspase of the extrinsic or death receptor mediated pathway), caspase 9 (early initiator caspase of the intrinsic pathway involving mitochondria) and combined activity of caspase 3 and caspase 7 (effector caspases activated by both pathways) was measured after exposure of RPMI8226 cells to TRAIL and compared with untreated cells. TRAIL was shown to be a potent activator of both extrinsic and intrinsic pathways (Fig. 5). In contrast, DOX activates only the intrinsic pathway and the level of activation is lower than the TRAIL-mediated activation of the same pathway.

### CD138^−^ cells are less sensitive to the cytotoxic activity of TRAIL, either alone or in combination with doxorubicin, when compared to CD138^+^ cells

**Figure 6:**
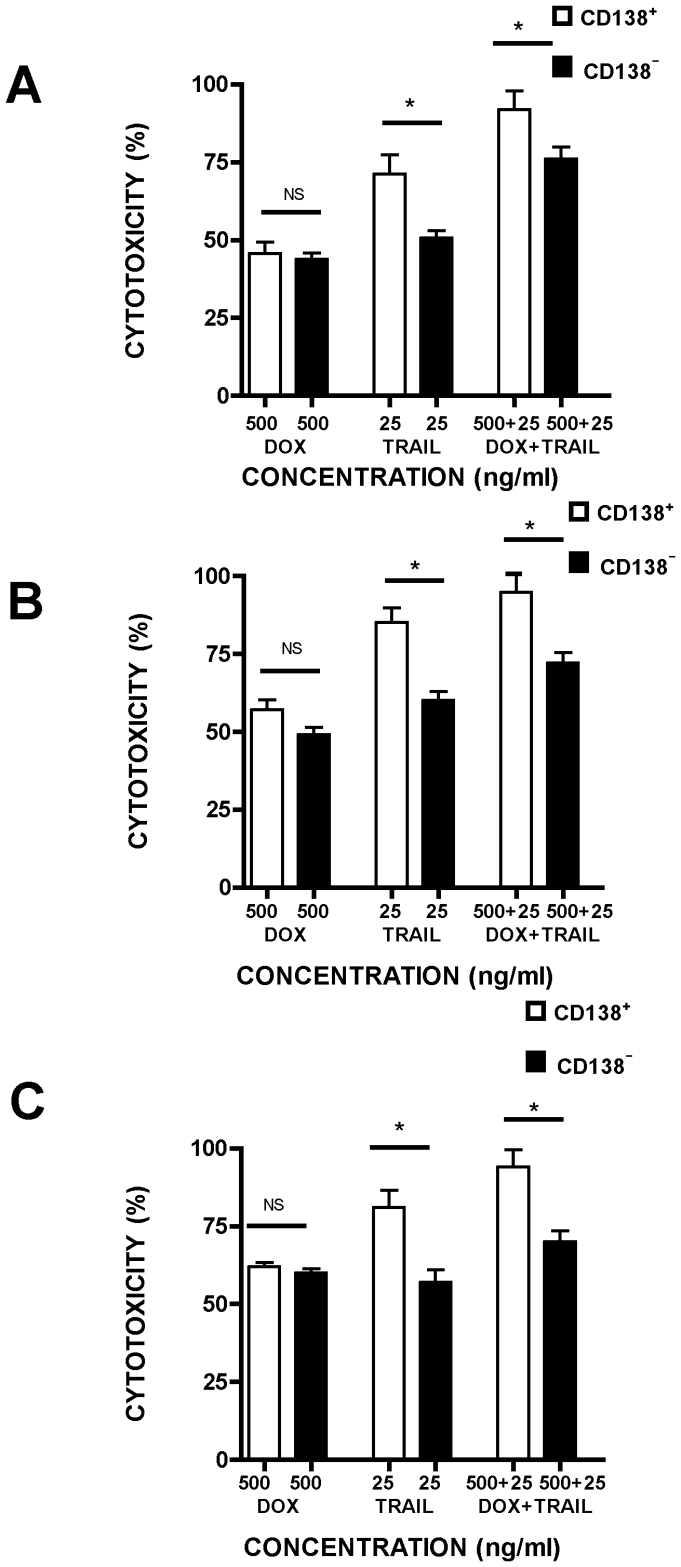
*In vitro* sensitivity of purified CD138^+^ and CD138^−^ myeloma cells to DOX and TRAIL alone, or TRAIL after pre-incubation with DOX. After immunomagnetic separation, CD138^+^ and CD138^−^ RPMI8226 (A), NCI H929 (B) and OPM2 (C) cells (1×10^5^ /ml) were incubated with DOX (500 ng/ml) and TRAIL (25 ng/ml) alone for 24 h, or TRAIL for 24 h after initial pre-incubation with DOX (500 ng/ml) for 24 h. Cytotoxicity was measured by MTS assay and expressed as a percentage of the untreated control sample. For details see Materials and Methods. Data represent the mean ±1SD of the three independent experiments.* = p<0.05

To determine whether the same sensitivity to TRAIL is present in the minor fraction of MM cells (2–3% of the total), we next compared the relative sensitivity of CD138^−^ and CD138^+^ cells. Cytotoxicity was measured after exposure of CD138^−^ and CD138^+^ RPMI8226, NCI H929 and OPM2 cells to TRAIL and DOX alone or after pre-incubation with DOX followed by TRAIL treatment (Fig. 6A, 6B and 6C). CD138^−^ cells were more resistant to the actions of TRAIL than CD138^+^ cells in each of the cell lines (p<0.01). TRAIL remained a potent inducer of cytotoxicity and this activity was further enhanced by pre-incubation of cells with DOX.

### Exposure of myeloma cells to TRAIL results in preferential killing of CD138^+^ compared to CD138^−^ cells

**Figure 7:**
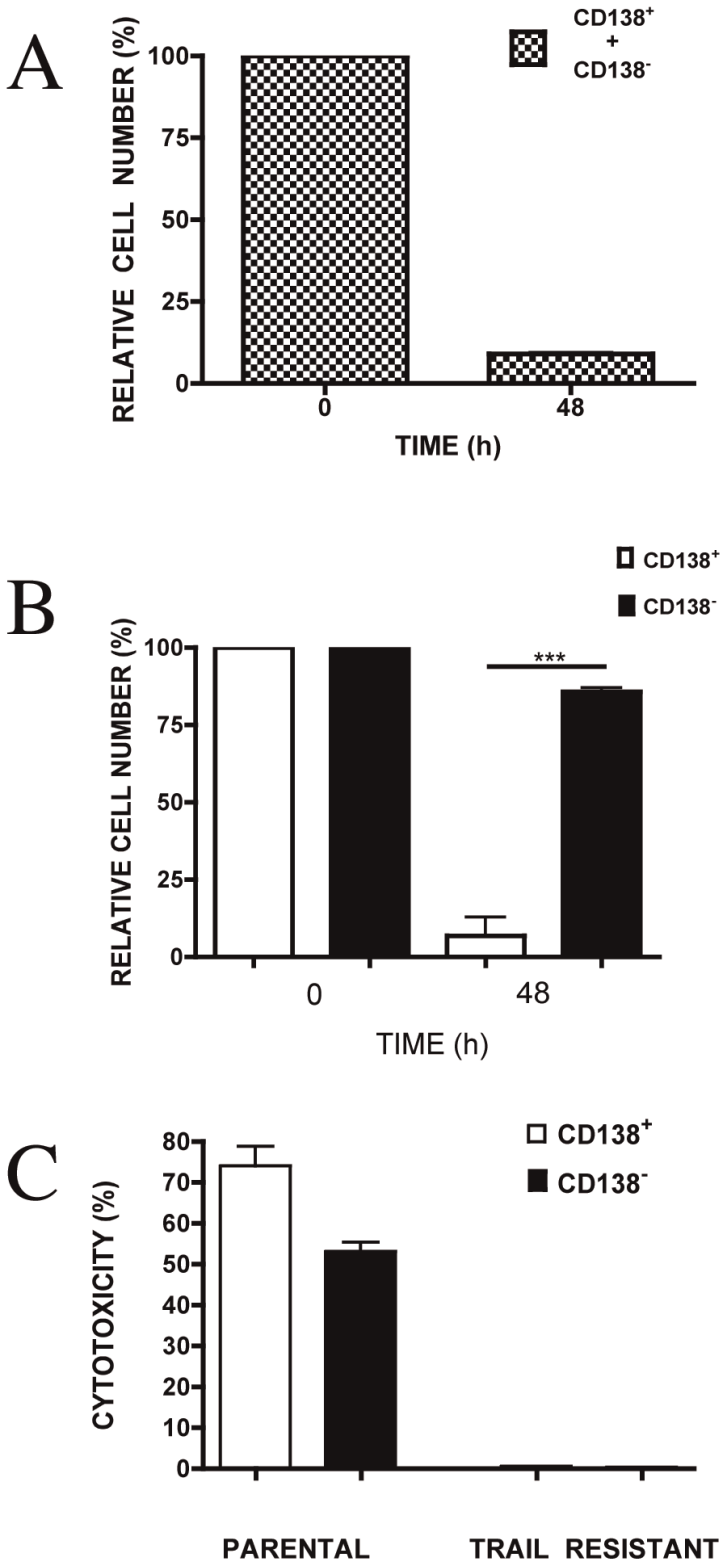
Relative difference in number of CD138^+^ and CD138^−^ RPMI8226 cells grown in culture, before and after exposure to TRAIL. (A) Relative total number (CD138^+^+CD138^−^) of cells in culture at the start of the experiment before the addition of TRAIL and after 48 h of incubation with 25 ng/ml TRAIL. (B) Relative number of CD138^+^ and CD138^−^ cells at the start before the addition of TRAIL and after 48 h of incubation with TRAIL. (C) Susceptibility of purified CD138^+^ and CD138^−^ cells isolated from TRAIL-resistant and parental, TRAIL-sensitive RPMI8226 culture. The susceptibility to TRAIL was determined using MTS assay and expressed as a percentage of the untreated control sample. Data represent the mean ±1SD of the three independent experiments. *** = p<0.001.

To determine whether reduced sensitivity of CD138^−^ cells to TRAIL has any relative survival advantage over CD138^+^cells, we investigated the relative level of reduction of CD138^+^ and CD138^−^ cell fractions in growing cultures of un-separated cells after exposure to TRAIL. Unsorted RPMI8226 culture was used in this experiment and treated with TRAIL (25 ng/ml) for 48 hours. One half of the culture prior to TRAIL exposure as well as the remaining TRAIL-treated half was used for immunomagnetic cell separation. After separation, the total numbers of CD138^+^ and CD138^−^ cells were determined before and after exposure of cultures to TRAIL. When exposed to TRAIL, the total number of cells (CD138^+^+CD138^−^) was reduced from 100% to 9±0.7% (91% reduction) (Fig. 7A). However, the number of CD138^−^ cells was reduced proportionally less than CD138^+^ cells: to 86±2.2% for CD138^−^ and to 7±10.4% for CD138^+^ (14% and 93% reduction respectively, p<0.0001) (Fig. 7B). This disproportional reduction in the number of CD138^−^ cells resulted in their relative increase from 3% to 45% of the total number of cells in the culture (initial ratio 1∶33 reduced to 1∶1.2).

### Prolonged incubation of MM cells with TRAIL results in the appearance of TRAIL-resistant CD138^−^ cells

The appearance of TRAIL-resistant cells has been reported previously, however the nature of this resistance, from the point of the existence of two distinct cell populations has not been investigated. We wanted to determine whether this resistance also appears in the CD138^−^ population. Therefore to facilitate the development of TRAIL-resistant culture we deliberately chose growth conditions, which favour the appearance of resistant cells (low concentration of TRAIL and prolonged exposure). Resistant RPMI8226 cells were obtained after continuous exposure of parental TRAIL-sensitive RPMI8226 culture to TRAIL (25 ng/ml, for 10 cycles of 5 days culture). Measurement of cell number and construction of growth curves showed that there was no significant difference between parental cells and TRAIL resistant cells (data not shown). Of these cells 96.25% were CD138^+^ and 3.75% CD138^−^. CD138^+^ and CD138^−^ cells from TRAIL-resistant culture were isolated using immunomagnetic separation and their susceptibility to TRAIL (25 ng/ml) compared to the parental, TRAIL-sensitive culture. CD138^+^ and CD138^−^ cells isolated from TRAIL-resistant culture were shown to be resistant to TRAIL (Fig. 7D).

### CD138^−^ cells have lower levels of expression of TRAIL receptor R1 and R2 than CD138^+^ cells

**Figure 8:**
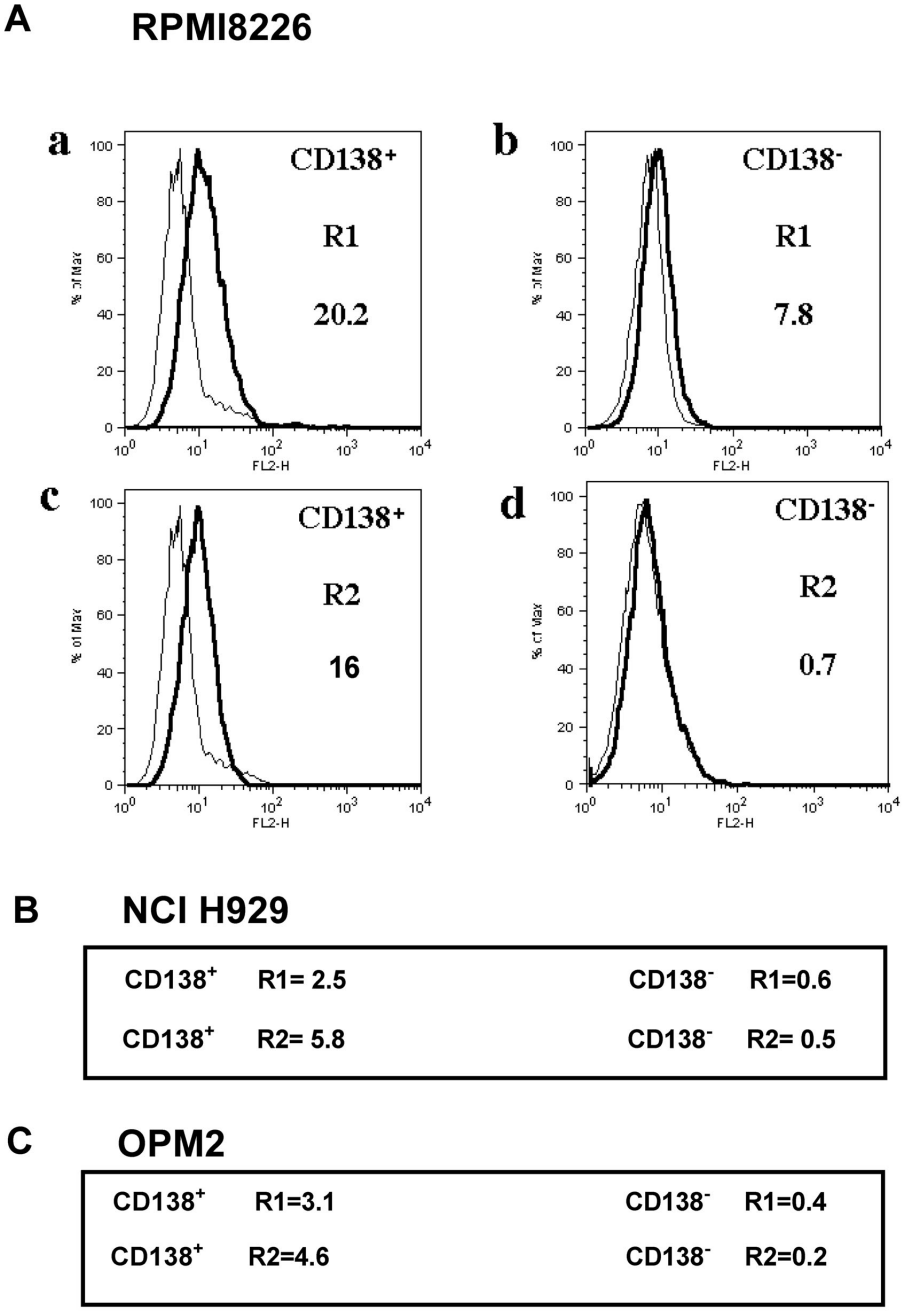
Expression of TRAIL receptors R1 and R2 on purified CD138^+^ and CD138^−^ RPMI8226, NCI H929 and OPM2 cells. Purified CD138^+^ and CD138^−^ cells were incubated with mouse anti-human TRAIL receptor R1 and R2 PE-conjugated monoclonal antibodies and analysed by flow cytometry to determine the percentage of labelled cells. The numbers indicate the percentage of receptor-positive cells. A, Representative histograms showing expression of R1 (a and b, thick solid lines) and R2 (c and d, thick solid lines) in CD138^+^ (a and c) and CD138^−^ RPMI8226 cells (b and d). The thin lines represent the appropriate isotype controls. B and C, Numerical representation of the percentage of CD138^+^ and CD138^−^ NCI H929 and OPM2 cells expressing R1 and R2 receptors.

To determine whether the differences in sensitivity to TRAIL were a reflection of differences in expression of TRAIL receptors, both CD138^+^ and CD138^−^ RPMI8226, NCI H929 and OPM2 cell populations were labelled with anti-TRAIL R1 and R2 PE-conjugated antibodies and analysed by a flow cytometry. FACS analysis revealed TRAIL R1 and R2 was expressed by CD138^+^ cells, although the level of expression varied between cell lines. A small proportion of CD138^−^ RPMI8226 cells were shown to express TRAIL R1 but not R2. CD138^−^ NCI H929 cells or OPM2 cells did not express TRAIL R1 or R2 (Fig. 8).

### Pre-incubation with DOX increases TRAIL sensitivity in both CD138^+^ and CD138^−^ MM cells isolated from patients with multiple myeloma

**Figure 9:**
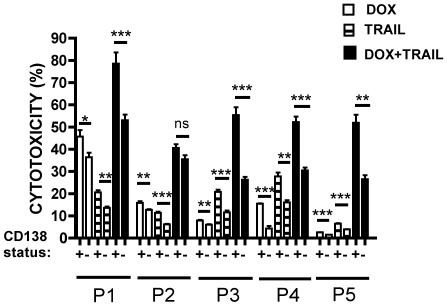
*In vitro* sensitivity of CD138^+^ and CD138^−^ CD34^−^ cells isolated from MM patient bone marrow samples. Sensitivity of CD138^+^ (+) and CD138^−^ CD34^−^ (−) cells from individual MM patient samples (P1–P5) to DOX (500 ng/ml, white bars), TRAIL (25 ng/ml, hatched bars) after 24 h and TRAIL for 24 h after pre-incubation with DOX for 24 h (black bars). Both arms of the study were time matched. Cytotoxicity was measured by MTS assay and expressed as a percentage of the untreated control sample. For more details about patient samples processing and cell purification please see Materials and Methods. Data represent the mean ±1SD of the three independent experiments. * = p<0.05, **p<0.01, ***p<0.001 and represent comparisons between CD138− and CD138+ cells.

The potential of TRAIL to induce apoptotic cell death in human CD138^+^ and CD138^−^ primary MM cells was investigated in myeloma cells isolated from newly diagnosed, previously untreated patients. Bone marrow samples from patients with newly diagnosed MM were used for these experiments. CD138^+^ cells were isolated by immunomagnetic separation. The remaining fraction was depleted of normal haemopoietic CD34^+^ cells to generate a CD138^−^CD34^−^ fraction. Both fractions were incubated with DOX or TRAIL alone for 24 h, and TRAIL for 24 h after pre-incubation with DOX for 24 h. DOX alone and TRAIL alone treated cells were also pre-incubated for 24 hours to match the length of treatment of DOX pre-incubated TRAIL-treated cells. Cytotoxicity was measured by MTS assay. The sensitivity of both CD138^+^ and CD138^−^CD34^−^ fractions was variable amongst individual patients samples (Fig. 9). The highest level of cytotoxicity was detected in cells treated with DOX and TRAIL, and this was seen in all samples. In all patient samples the cytotoxicity induced by the combination of DOX and TRAIL was greater than the sum of individual agents alone. This applied to both CD138^+^ as well as CD138^−^CD34^−^ cells (p<0.05). In all samples the CD138^−^CD34^−^ cells were less sensitive to the actions of DOX and TRAIL alone, or the combination DOX with TRAIL, than CD138^+^ cells (Fig. 9).

### Combined treatment with TRAIL and DOX results in complete eradication of plasmacytomas derived from CD138^−^ cells in NOD/SCID mice *in vivo*

**Figure 10:**
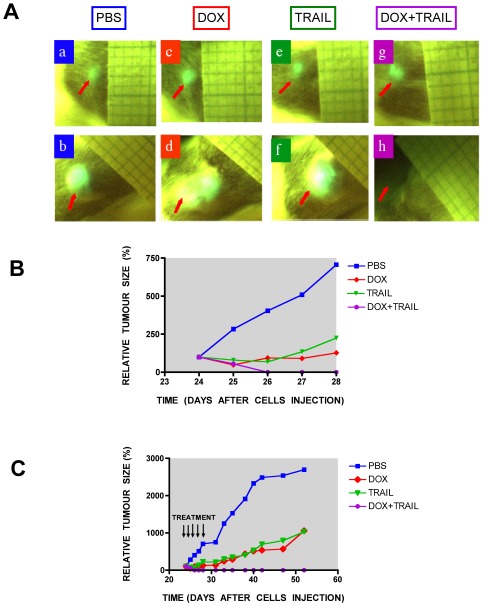
Complete eradication of established multiple myeloma plasmacytomas by combination therapy with TRAIL and Doxorubicin. (A) Representative images of mice before the start of the therapy at day 24 (a, c, e, g) and three weeks after the end of treatment (b, d, f, h). NOD/SCID mice were injected *s.c.* with CD138^−^ RPMI8226-GFP cells and tumours allowed to develop. Three weeks after inoculation, when the tumours were palpable, mice were randomised into four groups and treated with PBS (a, b), DOX (c, d), TRAIL (e, f) or DOX+TRAIL (g, h) for five consecutive days. DOX (1 mg/kg), TRAIL (10 mg/kg) and TRAIL+DOX (10 mg/kg daily+1 mg/kg on first and third day only) were administered *s.c.* Tumour size was monitored every day during the treatment week and every second day for an additional three weeks using LIGHTools imaging. For mice treated with PBS, DOX or TRAIL alone mice tumours continued to grow following cessation of treatment and reached a size requiring sacrifice. For mice treated with DOX and TRAIL tumours never reappeared so were studied for at least 90 days before being sacrificed. (B) Graph representing the mean tumour size for each group at a given day during the treatment period only. (C) Graph representing the mean tumour size for each group at a given day during the entire experiment. Graphs were constructed by digital analyses of tumour pictures. Data represent the mean for individual groups at each time point-SD data was omitted to improve the clarity of the final graph.

To determine whether TRAIL (alone or in combination with DOX) is also effective at inducing anti-myeloma activity *in vivo* we used the NOD/SCID mouse xenograft model of subcutaneous plasmacytoma growth initiated by implanting separated MM CD138^−^ cells (Fig. 10). Mice were first irradiated and 24 hours later CD138^−^ RPMI8226-GFP cells were injected subcutaneously into the right flank. After three weeks mice developed palpable tumours, were randomised into four groups and treated each day for five consecutive days with subcutaneous injections of vehicle, TRAIL and/or DOX. The first control group received PBS only, the second received DOX and the third TRAIL. The fourth group received DOX on the first and third day only and TRAIL on each day (Fig. 10A). Treatment with TRAIL or DOX alone, for 5 days resulted in a temporary inhibition of tumour growth (Fig. 10B), however, long-term follow up demonstrated that tumour growth continued once treatment had stopped. Thus using this regimen treatment only delayed tumour growth (Fig. 10C). However, the sequential treatment of DOX followed by TRAIL resulted in complete eradication of tumours (Fig. 10C). Indeed, we could not detect the presence of tumour even 90 days post treatment. Mice were sacrificed at this time and individual organs examined for evidence of tumour growth. No evidence of tumour growth was detected. No observable toxicity or weight loss was observed in this group.

## Discussion

In this study we report that TRAIL can induce apoptosis of different populations of myeloma cells. Importantly we show that TRAIL treatment in combination with doxorubicin results in the complete and sustained eradication of MM cells *in vivo*. Furthermore, we provide evidence that this treatment successfully eliminates CD138^−^ cells, which have been implicated in initiating myeloma growth [16].

In the present study we have shown that both CD138^+^ and CD138^−^ cells, derived from either cell lines or from patient samples, are susceptible to the actions of TRAIL, DOX and DEX. Of note these cells were more susceptible to TRAIL than either DOX or DEX. TRAIL when combined with DOX was shown to have a more profound cytotoxic activity in both populations than either agent alone. This is consistent with DOX upregulating/reinstating TRAIL sensitivity in these cells [25], [36]. We also provide three lines of evidence suggesting that apoptosis is the predominant mechanism of cell death induced by TRAIL. Cytospin experiments demonstrated evidence of morphological changes typical of apoptosis. Annexin V staining indicates changes in cytoplasmic membrane typical of early apoptosis. Finally, TRAIL was shown to induce caspase activity and induce apoptosis via both extrinsic and intrinsic pathways. In contrast our data suggests that DOX was unable to activate the extrinsic pathway, and is only a weak inducer of the intrinsic pathway.

We also demonstrated that CD138^+^ cells were more susceptible to TRAIL than CD138^−^ cells. This was seen in the human myeloma cell lines RPMI8226, NCI H929 and OPM2 as well as in cells isolated from patients with multiple myeloma. Although the magnitude of response may differ this could reflect the relative sensitivities of the different techniques used. The CD138^−^ cells have been reported to have tumour-initiating properties and support tumour growth *in vivo* [16]. Our studies also showed that prolonged exposure of cells to TRAIL results in the appearance of TRAIL resistant CD138^−^ cells. This increased drug resistance appears to be an important characteristic of cancer “stem” cells [22], [42]. This would argue that treatment in an *in vivo* or clinical setting should be short-term in nature in order not to induce drug resistance. Importantly, pre-treatment with doxorubicin and subsequent exposure to TRAIL resulted in increased ability to reduce viability of CD138^+^ and CD138^−^ cells *in vitro*. This was seen in each of the cell lines examined. The source of the CD138^+^ cells in these cultures is unknown. Whether they are derived directly from CD138^+^ cells or arise form the CD138^−^ population is unclear.

In primary myeloma cells TRAIL was also able to induce cytotoxicity. This was more pronounced in CD138^+^ when compared to CD138^−^CD34^−^ cells. Since this population may contain the myeloma-initiating cells [16] our data suggest that the clonogenic progenitor cells, whether they are in cell lines or derived from patients, are less susceptible to the actions of TRAIL. However, as with the cell lines combined treatment with TRAIL and DOX significantly reduced the growth of CD138^−^CD34^−^ cells. Unlike with CD138^−^ cells present in the RPMI8226, NCI H929 and OPM2 cells combined treatment at the concentrations used did not eradicate growth of primary myeloma cells *in vitro*. The reason for the difference in sensitivity between primary cells and the cell line cells is unclear, but may reflect differences in levels of TRAIL receptors and/or downstream signalling components between samples and cell lines. Certainly, our data demonstrated differential expression of levels of TRAIL R1 and R2 amongst the cells lines. To eradicate primary tumour cells completely, further studies establishing optimum dosing regiments may be necessary.

Having demonstrated that treatment of TRAIL with DOX was able to eradicate CD138^−^ cells *in vitro* we then investigated this *in vivo*. Given that CD138^+^ and CD138^−^ were shown to be able to develop resistance to the actions of TRAIL we chose to use short-term treatment only. Once solitary plasmacytomas had been established mice were treated for five days with TRAIL and/or DOX and monitored for up to 90 days following treatment. Using this treatment regime and a native TRAIL preparation rather than forms of TRAIL used previously with FLAG, polyhistidine or leucine zipper tags [35], [40], we demonstrated that treatment with TRAIL alone resulted in a rapid initial reduction in tumour size. This reduction was only transient and further increase in tumour size was apparent even during the treatment phase. The overall result of this treatment was only to delay tumour growth. Similar results were obtained with DOX only treatment. However, if TRAIL was combined with DOX pre-treatment it was possible to achieve total tumour eradication, using both fluorescence and visual analysis, in all animals. This is consistent with reports that DOX enhances sensitivity to TRAIL by multiple mechanisms. Previous studies have shown that DOX potentiates the effect of TRAIL by inducing expression of TRAIL death receptors DR4 and DR5 [36]. DOX may further enhance TRAIL mediated apoptosis through induction and activation of pro-apoptotic caspases and down-regulation of the inhibitors of the apoptosis family of proteins (IAP) [43], [44]. DOX also induces expression of Smac/DIABLO, a suppressor of IAP, and ceramide production, which enhances DR5 clustering in the cell membrane [45], [46]. We could not detect any tumour growth in animals treated with a combination of DOX and TRAIL either in live mice or following post-mortem analysis at 90 days, either following visual analysis or fluorescence analysis. Although we cannot exclude the possibility that tumours were present but too small to be detected using these techniques it does support the suggestion that combination therapy is able to cause a sustained eradication of the tumour *in vivo*. We conclude, that as with combination treatment *in vitro*, combination treatment *in vivo* is able to eradicate both CD138^+^ and CD138^−^ cells. Importantly, the short-term nature of the treatment and the combination with DOX prevents the development of resistant cells. These findings may provide a rational explanation for the failure of TRAIL alone to eradicate tumours. This may have implications for treating cancer in general. For example, it has been reported that mouse mammary tumours, treated with chemotherapeutic agent cisplatin, become more resistant to further treatment and contain proportionally more cancer “stem” cells than responsive tumours [47]. High frequencies of leukaemia stem cells have also been detected in poor-outcome childhood precursor-B acute lymphoblastic leukemias [48].

The anti-cancer potential of TRAIL (alone or in combination) has been demonstrated in various *in vivo* models of tumour growth. Colon, breast, prostate, glioma and MM tumours grown in mice have all been shown to be susceptible to TRAIL [29], [30], [38], [49], [50], however the reported efficacies of these treatments were variable. This may reflect many factors including the design of the experiment, the molecular form of the TRAIL protein used, the appearance of resistant cells, the duration of treatment and the length of tumour monitoring after cessation of treatment. For example, the efficacy of treatment for prevention of tumour establishment and growth was higher than for treatment of established tumours [30], [49]. Equally, the bigger the established tumour prior to treatment, the less efficacious the treatment [30]. It has also been shown that preparations of TRAIL containing non-native domains and/or requiring antibody for its polymerisation are less efficient than the native form of TRAIL, at least in *in vitro* experiments [51]. Extending the duration of treatment may increase efficacy but also increases the probability of appearance of resistant cells [50]. Data from clinical studies suggests that similar processes might operate during treatment. Certainly treatment of patients with myeloma usually involves administration of chemotherapeutic drugs over a period of weeks or months. The initial treatment successfully reduces tumour burden, however, after a period of time, patients relapse which often results in the development of more resistant disease, which are increasingly difficult to treat. However, our data would argue that for optimal use of TRAIL it is important to consider using short-term treatments and combining this with agents that may sensitise the tumour cells to the action of TRAIL.

In summary, our data suggest that it is possible to eradicate myeloma cell growth *in vitro* and in a model of solitary plasmacytomas *in vivo* by targeting CD138^−^ myeloma cells with TRAIL. Importantly, combining short-term TRAIL treatment with DOX prevents the development of TRAIL resistant cells, especially in clonogenic progenitor population, and the long-term recovery of the tumour *in vivo*. We believe that the findings presented here offer a promising new approach in the quest to cure disseminated cancers such as MM.
